# Construction Notes

**Published:** 1895-07-20

**Authors:** 


### CONSTRUCTION NOTES.

### Women's and Children's Hospital, Cork.

Some time ago the committee of the above hospital, anxious to provide the institution with every modern appliance, decided on procuring for it a patients' lift, the order for which was placed in the hands of an eminent London firm. The lift has now been completed. It is constructed on the suspended principle, in which the cage is raised by ropes, and in this case there are four crucible steel wire ropes of more than sufficient strength to sustain the full load alone. The cage is of substantial and neat appearance, and will accommodate a patient in bed and the nurses attending. It has been manufactured by Messrs. R. Waygood and Co., of Falmouth Road, London.

### Passmore Edwards Cottage Hospital, Liskeard.

One of the foundation-stones of the Liskeard "Passmore Edwards" Cottage Hospitals, the first of the series of hospitals and libraries Mr. Passmore Edwards is inaugurating in Cornwall, has been laid recently, according to Masonic honours, by Lord Mount Edgcumbe, Provincial Grand Master of Cornwall. The building is intended for the reception of a moderate number of free patients, and also for people from outlying districts, who would pay, and thereby help to support the institution. The building is also intended as the headquarters of parish nurses, an institution of considerable growth in the West of England. The site was given for the hospital by Mr. Marshall. The hospital is built of stone, with brick dressings; the internal woodwork is of pitch pine. When built the institution will be taken over and managed by the Town Council of Liskeard and by a committee; it is the gift of Mr. Passmore Edwards. The designs show the administration block to be situated in the centre of the building and wings on the right and on the left. One of these will contain a ward for three men, and the other a ward for three women, added to which there is to be constructed an accident ward. In addition to these there are to be two smaller wards, which, when not otherwise used, will be for private patients. A special feature of the building is the provision in the centre block for three bed-rooms and sitting-rooms for the nurses of the Liskeard district. All the sanitary, warming, and lighting arrangements will be of the latest and most approved order. The work is being carried out by Messrs. J. Symonds, of Blackwater, from the designs of Mr. J. Hicks, architects, of Redruth.

